# A Debate on Cerebro-Spinal Meningitis: The Problems of Military Epidemics

**Published:** 1915-03-06

**Authors:** 


### A DEBATE ON CEREBRO-SPINAL MENINGITIS.

### The Problems of Military Epidemics.

The occurrence at the present time of several outbreaks of cerebro-spinal meningitis is attracting a very considerable amount of attention in medical circles. This was plainly shown on Friday evening last week, when there was an exceptionally large attendance at the meeting of the Epidemiological Section of the Royal Society of Medicine. Owing to the fact that in certain localities cases of this meningococcal infection have occurred among our soldiers, the military element was exceedingly well represented in the audience which assembled to hear Sir William Osler open the special discussion on the epidemiology of this disease.

Limitation of time and the number of would-be speakers prevented the discussion from being concluded the same evening, and it had perforce to be adjourned for a week before many of the more practical aspects of the subject had received anything like adequate attention—a circumstance that was plainly disappointing to the officers of the R.A.M.C., many of whom had come long distances to pick up valuable information to be used for the benefit of urgent cases now under their care.

Nevertheless, the evening was far from being unprofitable. Sir William Osler introduced the subject in a scholarly address, which dealt more particularly with the natural history of the disease, the difficulties in diagnosis, and the prognosis of this infection, which is more hopeful than that of the other meningitides. Though the case mortality of cerebro-spinal meningitis is so high, this "infectious fever" accounts for far fewer deaths, as was pointed out by Dr. Newsholme, than many other specific infections such as pneumonia or measles. The experience of Dr. A. K. Chalmers, the medical officer of health for Glasgow during the epidemic which occurred in that city seven or eight years ago, furnished some interesting information on the behaviour of this disease from the epidemiological point of view. Its well-known propensity to attack single members only of a household was illustrated by the fact that in Glasgow in only 4 per cent. of the houses was there more than one case.

The influence of overcrowding on the spread of cerebro-spinal meningitis is direct, and is conceded by all observers to have greater influence than either personal or environmental filthiness.

The question of "carriers" was touched on by all who shared in the debate, and it is certain that the subject will receive more attention when the adjourned meeting takes place. It is universally admitted that carriers of the meningococcus present a very difficult problem in connection with the administrative action called for on the outbreak of epidemics of cerebro-spinal meningitis. In one epidemic it was found that 26 per cent. of the "contacts" were carrying the meningococcus, and in another instance, in the South of England, two cases of cerebro-spinal meningitis gave rise to no fewer than twenty-four carriers. It cannot be doubted that these healthy carriers actually disseminate the disease, although, as in the case of the Klebs-Löffler bacillus of diphtheria, it is somewhat rare for the organism to produce the disease in the carrier. On the occasion of the visit of the Prince of Wales to Glasgow in 1907, a marked increase in the number of cases of cerebro-spinal meningitis occurred during the following week, and no doubt carriers in the crowds were to a large degree responsible for this.

The lurking place of the meningococcus is the upper part of the naso-pharynx—a situation which offers not only difficulties in the taking of swabs, but also affords the microbe very efficient protection against any destructive measures directed against it. In the taking of swabs it is most important to avoid contamination of the swab by the bacteria of the fauces. Swabs taken, as in diphtheria, from the fauces are of little or no value. In swabbing the naso-pharynx for the meningococcus it is necessary to use not only a suitably curved metal rod, but also one which has a protecting cannula. The swab is mounted on a long, curved, rigid wire, and this lies within the cannula until the uvula has been passed, when the swab is extruded. After careful rubbing of the swab over the mucous membrane of the naso-pharynx, the rod is withdrawn into the cannula, and, thus protected, is removed from the mouth. The difficulties of bacteriological examination are not even now at an end, for the meningococcus is not a hardy organism. It will not live long on the swab, so it is highly desirable that the coccus should be sown on suitable media at once, preferably on a Petri dish, which should be forwarded to a laboratory without delay. In actual practice it is advisable to inoculate a second Petri dish from the first to avoid overcrowding. It will readily be realised that swabbing for the meningococcus is apt to prove valueless unless done by an expert, and that negative results may often be of no value whatever, especially when ordinary swabs are sent by post to a laboratory.

When found residing in the naso-pharynx the meningococcus has proved exceedingly resistant to all measures which have been directed against it. Antiseptics of various kinds, whether liquids or vapours, have proved totally unreliable. Some hope is entertained that a vaccine will prove efficacious in ridding a carrier of these organisms.

In public-health circles an increase of the incidence of cerebro-spinal fever is regarded as very probably imminent, and owing to the fact that the virulence of the infecting organism tends to become reinforced during epidemics, it is to be expected that cases of the fulminant type will occur in fair numbers. These cases, in which death ensues very rapidly, not rarely escape recognition, and may be mistaken for other forms of meningitis, or, indeed, typhus fever, malignant measles, tetanus, or pneumonia. Hence administrative action as regards contacts becomes of great importance. All persons who have been attending or personally associated with a case of cerebro-spinal meningitis ought to be kept under observation as possible carriers of infection. Nothing dogmatic can be stated as to the duration of the infectivity of contacts, but the very recent Memorandum issued by the Local Government Board suggests as a useful rule that contacts should be regarded as possibly infective for three weeks from the date of last association with a case. An open-air life for contacts is strongly recommended, and the isolation of such contacts in hospitals is to be deprecated.

The practical subject of treatment was not given too prominent a position at the meeting, for the section is primarily concerned with epidemiology, but naturally the question of the value of serum treatment was touched upon by several speakers. Undoubtedly there is much yet to be learned concerning the efficacy of the serum, whether Flexner's serum or those brands made in this country are under consideration. Certainly it was made to appear that Flexner's serum had in some hands produced almost revolutionary changes in the death-rate. In the experience of one speaker the mortality fell from between 72 and 82 per cent. to something like 25 per cent. in four or five months. It is also interesting to note that during a certain period only about half the cases were sent to hospital, and of thirty-four who preferred to remain at home (and thus did not receive any serum treatment) no fewer than twenty-nine died, while of thirty-four sent to the hospital, who received the interspinal injections of serum, only eight died. The results of serum treatment are, however, not yet uniformly good, but it is to be hoped that the serum now prepared by the Lister Institute will show even better results than Flexner's serum, which seems to have been decidedly the most successful (and most used) so far.

